# Delayed Onset of Motor Blockade After Liposomal Bupivacaine Use for a Perioperative Popliteal Nerve Block: A Case Report

**DOI:** 10.7759/cureus.24031

**Published:** 2022-04-11

**Authors:** Sameer Shah, Raffy Mirzayan, Johannes Bernbeck, Yukta Italia, Atef Morkos, Chunyuan Qiu, Vimal Desai

**Affiliations:** 1 Research, University of California Riverside, Riverside, USA; 2 Orthopedics, Baldwin Park Medical Center, Kaiser Permanente, Baldwin Park, USA; 3 Orthopedics, Los Alamitos Medical Center, Los Alamitos, USA; 4 Research, University of California Los Angeles, Los Angeles, USA; 5 Anesthesiology, Baldwin Park Medical Center, Kaiser Permanente, Baldwin Park, USA

**Keywords:** delayed onset, exparel, liposomal bupivacaine, adductor nerve block, popliteal regional nerve block

## Abstract

We present a case of a 60-year-old male who has undergone Achilles’ tendon repair with amnion augmentation on the right side. Before the surgery, liposomal bupivacaine was requested by the patient, and this was used to block the popliteal and adductor canal nerves for intraoperative anesthetic and postoperative pain control.* *The patient understood the benefits and risks of the regional nerve block with this medication and its off-label use. After the surgery, the patient underwent an irregular course of anesthetic, including delayed motor weakness, and became non-weight-bearing temporarily. The motor block was inconsistent and non-linear. Initially, the motor block completely resolved with a return to the sensation after three days. Then, on day four, a complete motor block developed, which resolved gradually over the next four days. After 10 days, the patient had full resolution of symptoms. He denies any pain since the surgery.

## Introduction

The use and predictability of sustained-release liposomal bupivacaine have been a subject of interest for regional acute pain anesthesiologists for some years [1,2]. As per a recent meta-analysis, compared with a local anesthetic, perineural liposomal bupivacaine provided no benefit and a clinically insignificant improvement in postoperative pain scores for peripheral nerve blocks [3]. Its applicability for surgical wound infiltration as with immediate-release bupivacaine HCL has been well sustained and is widely practiced [4]. Its effect on postoperative pain control for a wide variety of procedural wounds is also FDA approved [5]. Its use for regional nerve blocks has only been FDA approved for nerve blocks such as the following: transverse abdominal plane block [5] and interscalene nerve block. Liposomal bupivacaine is a sustained release and a long-acting local anesthetic [6]. Its off-label use safety is difficult to ascertain, and cases seldom arise [7]. This case report can provide guidance and risk-benefit analysis.

We report a case showing the effects of blocking the adductor canal and popliteal nerves. This patient received the block preoperatively. After 30 minutes, he had a sensory blockade of the popliteal and adductor nerve distribution, but intact motor function. After the procedure was done, the patient described a similar exam with complete pain relief.

## Case presentation

A 60-year-old male with a history of basal cell carcinoma (location unknown), allergic bronchitis, cervical radiculitis without current neuropathy, and history of sciatica with laminal decompression and no residual neuropathy presented with pain in the right ankle. His past surgical history was significant for the history of total hip replacement, lumbar laminectomy, and repair of the rotator cuff.

On examination, this 6’ 4’’, 93 kg male had a mild right ankle swelling, and intact sensation to light touch L5-S1/C5-T2. His right ankle was tender to palpation along the course of the Achilles’ tendon with palpable depression. His range of motion at the right ankle joint showed full dorsiflexion and guarded plantar flexion. Thompson’s test was positive. Neurological and vascular examinations were normal. His MRI of the right ankle showed Achilles’ tendon rupture. He underwent repair of the right Achilles’ tendon. The patient's anesthetic consisted of general anesthesia with versed and fentanyl preoperatively and propofol for induction. The patient was maintained under anesthesia with sevoflurane anesthetic gas. The patient did not require paralysis. The patient's vitals and intra-operative course were uneventful. He had a fully functional range of movement after surgery.

The patient had popliteal and adductor canal blocks for Achilles’ tendon repair. Liposomal bupivacaine (Pacira Bioscience Inc., Parsippany, NJ) was utilized. For the popliteal nerve block, we utilized 15 ml of 1.3% Bupivacaine Liposome Injectable Suspension (199.5 mg) mixed with 5 ml of 0.5% bupivacaine (25 mg). We used 5 ml of 1.3% liposomal bupivacaine (66.5 mg) mixed with 5 ml of 0.5% bupivacaine (25 mg) for the adductor canal block. The patient requested the use of liposomal bupivacaine, and the risks and benefits were discussed in detail. Although the patient has had nonlinear sensory and motor loss pattern, he has had minimal to no pain since his surgery (Table 1). The patient reported no sharp, stabbing, burning, or incisional pain but did report minor tolerable pressure discomfort. In Table 1, we recall the day-to-day neurological exam, which was assessed and reported by the patient through phone visits with the attending anesthesiologist on a day-to-day basis.

**Table 1:** POD 0 to 10, the daily course of patient's sensory, motor, and pain scores POD: Post-operative day

POD	Sensory Function out of 5	Motor function out of 5	Pain Score out of 10	Other information
0	0	4 at toe dorsiflexion	0	Ankle in splint
1	0	4 at toe dorsiflexion	0	Ankle in splint
2	1	4 at toe dorsiflexion	0	Ankle in splint
3	0	5 at toe dorsiflexion	3	Ankle in splint
4	0	0 entire foot	0	Splint removed, pressure ulcer on the shin (stage 2)
5	0	In the morning, 2 at toe dorsiflexion In the afternoon, 0 at toe dorsiflexion	0	-
6	0	2 at toe dorsiflexion	0	-
7	3	3 at toe dorsiflexion function, 5 elsewhere	0	-
8	4	3/5 at toe dorsiflexion (motor function otherwise normal)	0	-
9	4	5 entire foot	0	-
10	5	5 entire foot	0	-

**Figure 1:**
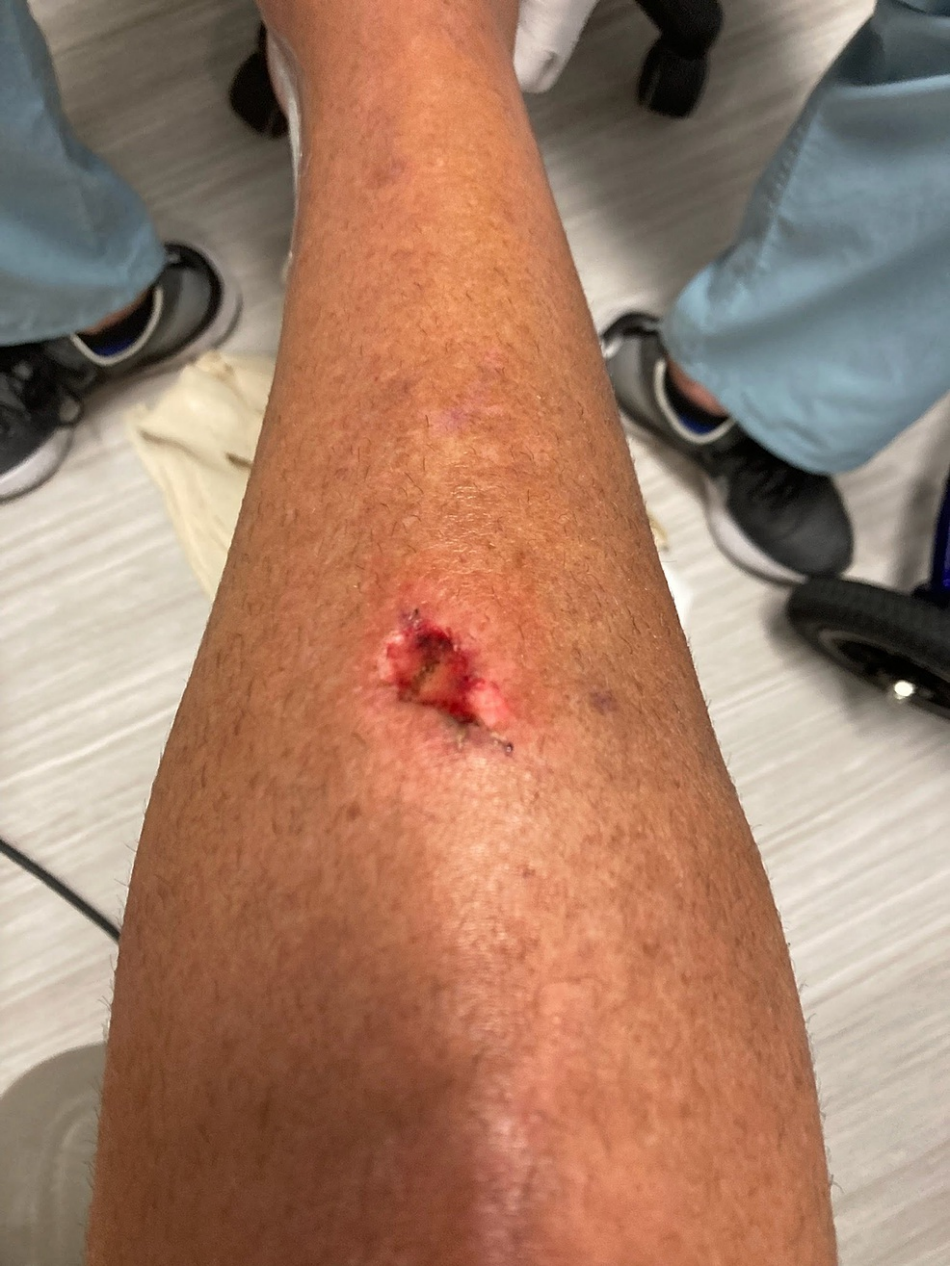
Image of the patient's stage 2 pressure ulcer on the shin

On POD 3, the patient reported some distress as there seemed to be a partial resolution of the block. On POD 4, he noticed the recurrence of the numbness and an increase in the density of the block with new-onset motor weakness. The motor block began to improve on POD 5 with full resolution on POD 8. Of note, the patient also developed a stage 2 shin ulcer due to the lack of protective sensation and persistent swelling on the heel (Figure 1). The patient said, “I never had any real pain and never had to take any pain meds, not even an acetaminophen.”

## Discussion

Pain-free procedures have been an endeavor of medical professionals; however, we seem to be knocking on the door of this reality throughout the perioperative timeframe [8]. Postoperative pain continues to be a difficult surgical outcome to manage in addition to chronic pain, centralization, and its associated costs [8]. Extended nerve blocks have been experimented with, which include the utilization of adjuncts, catheters, improved block techniques, and sustained release local anesthetics [9,10]. The reliability and predictability of these various techniques continue to be problematic while the use of the unmodified local anesthetic, such as bupivacaine, remains the most predictable. However, its immediate-release form does not cover the perioperative postoperative pain period which can range from three to five days [11]. Liposomal bupivacaine, which has been FDA approved for postoperative pain control, is a sustained release and long-acting local anesthetic [12]. Liposomal bupivacaine duration of action is up to 72 hours. Plasma levels of liposomal bupivacaine can persist for 96 hours after local administration and 120 hours after interscalene brachial plexus nerve block [13]. 

In our case report, we find that the patient’s pain was well controlled for the perioperative period with liposomal bupivacaine mixed with a small dose of immediate-release bupivacaine. The patient sustained 0/10 pain and had a block duration of about 8 days. The scale used was a numerical rating pain scale with 10 being the worst pain the patient ever felt. However, the motor block was inconsistent and nonlinear. It began with a mild deficit of 4/5 on the day of the block and continued postoperative day POD#2 with complete resolution on POD#3. However, on POD#4 there was a complete motor block which resolved gradually over the next four days. A neurological exam was conducted through a phone call visit with clear instructions to assess a motor and sensory exam; however, this comes with its own self-assessment bias. It should also be noted that there should be caution with prolonged blocks since there is a risk of unrealized injury or ulcer.

The mechanism of this pattern could be secondary to the delayed release of bupivacaine from the liposomal capsules. It is hypothesized to be secondary to mechanical release, possibly from physical therapy onset, increased range of motion, or changes in pH of unknown origin. It should be noted that no local anesthetics were injected by the operating surgeon to lead to inconsistent release.

## Conclusions

The use of liposomal bupivacaine for popliteal and adductor canal blocks for sensory blockade throughout the postoperative period is promising. As with any regional block and more so with a prolonged block, there must be precaution as to the possibility of injury due to lack of sensation and visualization as well as care of the affected area. This is a very effective pain control strategy, but extra caution should be taken to assess the skin and to avoid pressure ulcers. In our patient, the inconsistency of the motor blockade may be common or uncommon, and further studies in a more controlled environment must be undertaken. It is vital for clinicians and patients to be conscious of its risks and inconsistencies during its use as outlined in this case.
